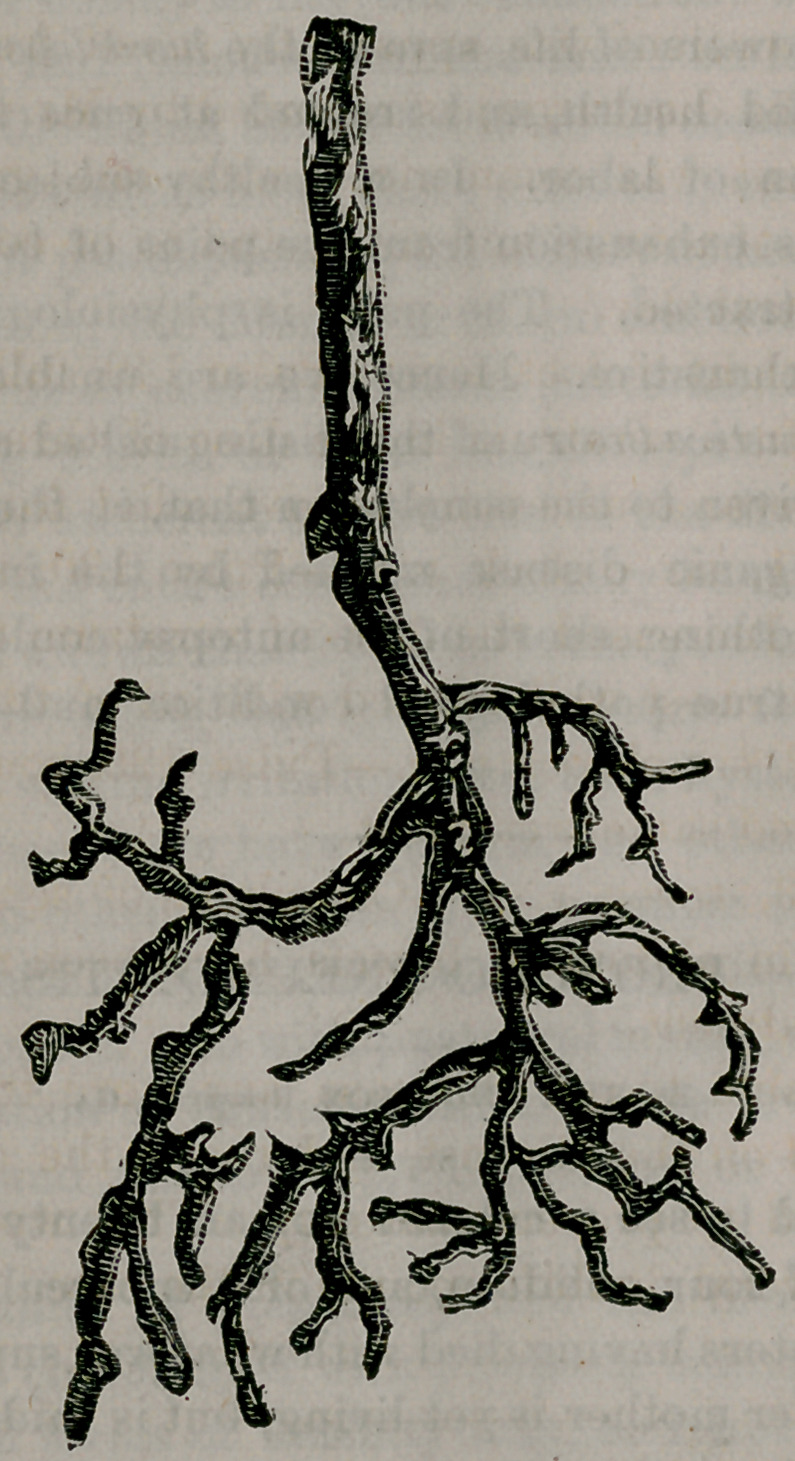# Broncho-Pneumonitis

**Published:** 1877-09

**Authors:** R. B. Anderson

**Affiliations:** Roswell, Ga.


### BRONCHO-PNEUMONITIS.

By R. B. ANDERSON, Roswell, Ga.

Was called to see a colored woman twenty-two years old, the mother of four children, and of a tuberculous family, several of her sisters having died with what was supposed to be tuberculosis. Her mother is yet living, but is said to be consumptive. I saw the patient first on the 30th of August, at 10.40 P.M., and found her suffering with great difficulty in breathing, requiring to be propped in bed, pain in the abdomen, cough, with bloody expectoration, very frequent, quick and small pulse. Bronchial sibilant rale was the prominent sound revealed by auscultation on both sides of the chest, with general dullness on percussion, all over both lungs.

I learned from Mr. Hook, an intelligent gentleman, whose father was former owner of the patient's family, that she had been complaining of diarrhœa for several days, and had frequent paroxysms of cough, with bloody expectoration. He had preserved, and presented to me, a clot which had been discharged during a fit of coughing, a short time before my arrival. This, on inspection, proved to be a complete cast of one of the bronchial trunks and its branches. He reported the patient on the point of suffocation before this was discharged.

Never having met with so interesting and rare pathological specimen, I had it photographed for the preparation of the accompanying cut. It had been slightly mutilated, perhaps before it came into my possession. This I very much regret, but the cast is sufficiently perfect to show distinctly a mould of the tube and ramifications. The trunk, in its flattened position, measures three-eights of an inch, and the arborescent formation of trunk and branches measures six inches in length, and three inches across the branches, when lying, as nearly as I can place them, in their natural position, after the loss of a portion.

Whether this is a pseudo-membrane, consisting of fibrinous formation, the result of diphtheritic inflammation, or the separation, entire, of the broncial mucous membrane, I am not able to decide. Certainly, however, we have an exact mould of the respiratory tubes in the specimen. The former appears more probable, and the distressing dyspnœa is readily explained on this hypothesis, particularly when it is remembered that the patient was apparently saved from immediate suffocation by the discharge, as reported by Mr. Hook.

The treatment was commenced with one grain doses of opium, tincture of aconite and nitre, and to be continued until my return the next day.

August 31, eight o'clock P. M.—Pulse 140, full and strong. Prescribed tincture veratrum in the dose of three drops, nitre and opium as became necessary to give relief from pain.

September 1, six o'clock P. M.—Pulse 130, some general improvement. Increased the dose of veratrum to four drops, and ordered the chest well rubbed with oil of turpentine.

September 2, six o'clock P. M.—Pulse 126, cough more frequent—continued same treatment.

September 3, seven P. M.—Complains of sense of suffocation, and pain in abdomen; bowels have been moved. Continue same treatment.

I was not able to see the case any more till her death, which occurred on the 4th inst, and regret the circumstances which conspired to prevent autopsy. This would certainly have settled several doubtful points in the case.

## Figures and Tables

**Figure f1:**